# A Test Method for Identifying Selection Bias Risk in Prospective Controlled Clinical Therapy Trials Using the I2 Point Estimate

**DOI:** 10.7759/cureus.60346

**Published:** 2024-05-15

**Authors:** Steffen Mickenautsch, Veerasamy Yengopal

**Affiliations:** 1 Dentistry, University of the Western Cape, Cape Town, ZAF; 2 Community Dentistry, University of the Witwatersrand, Johannesburg, Johannesburg, ZAF

**Keywords:** clinical trial appraisal, selection bias, i2, clinical trial, bias testing

## Abstract

Objectives: A test method is proposed for identifying potential selection bias risk in single prospective controlled clinical therapy trials that can be applied by trial reviewers.

Methods: The method is described in detail and was tested on eight randomised controlled trials (RCTs) with reported negative Berger-Exner test results as negative and on eight prospective, controlled cohort studies as positive controls. All 16 studies were identified by systematic literature search.

Results: The test method yielded negative results for all RCTs and positive results for six out of the eight cohort studies.

Conclusion: All test results remained within the expected limits for both study types, suggesting a reasonably high accuracy for correctly identifying selection bias risk. However, the method does not provide the possibility to establish whether such bias risk has actually altered trial outcomes. Instead, a positive test result may provide an empirical basis for rating a trial as of high selection bias risk during trial appraisal.

## Introduction

Randomised control trials (RCTs) are considered the gold standard of effectiveness research for clinical therapy [1]. However, even if reported to have followed a flawless randomisation procedure, RCTs may still carry a high selection bias risk. Current trial appraisal tools such as the second version of Cochrane’s Risk of Bias tool (RoB 2) [2] or the latest version of the Composite Quality Score (CQS-2B) [3] are methods that can only scan trial reports for possible indicators of systematic error in the text. They are unable to quantitatively analyze whether high bias risk actually exists.

In response to the need for such quantitative tools, Berger and Exner developed the Berger-Exner test for detecting third-order selection bias in RCTs [4]. The test comprises linear regression analysis, conducted separately per treatment group, with the reverse propensity score (the propensity of a patient being allocated to one of the intervention groups) as independent and the patient’s trial outcome value as a dependent variable. The accuracy of the test has been established as being very high with a test sensitivity of 1.00 (95% CI: 0.99-1.00) and test specificity of 0.94 (95% CI: 0.93-0.96) for alpha set at 1% [5]. However, the test has the disadvantage that it can be conducted only on the basis of individual trial patient data, which is seldom published as supplementary material to RCT reports. Therefore, the test can in most cases be applied by only the trial authors themselves and not by trial reviewers during, for example, the conduct of a systematic review of clinical trials. Hence, the need for more useful bias tests remains.

In 2014, Hicks et al. suggested that, because the true random allocation of patients in RCTs ensures a balanced distribution of baseline characteristics in intervention groups, heterogeneity in baseline variables should always be zero and any measured differences in baseline values between the groups could occur only by play of chance [6]. Clark et al. stated that baseline variables, common to all trials in a meta-analysis, do not share explanations for heterogeneity in outcome variables (such as populations or intervention differences) and that the only plausible explanation for heterogeneity in baseline variables is poor randomisation [7].

The lack of heterogeneity in baseline variables is reflected by a zero I^2^ point estimate in a baseline data meta-analysis. The I^2^ point estimate ranges between 0 and 100% and was originally developed for the purpose of estimating the proportion of variance in trial outcome estimates that are due to heterogeneity between trials rather than chance [8]. However, when the I^2^ point estimate is used in a baseline data (instead of an outcome) meta-analysis, baseline imbalances of one or more trials caused by non-random allocation of patients to intervention groups will deviate from a zero value and thus indicate that the meta-analysis result is affected by selection bias [7]. On this basis, Hicks et al. presented a simple technique for identifying and eliminating potential bias in meta-analyses [6].

Based on the same principles outlined above, a test method is proposed for identifying potential selection bias risk in single prospective controlled clinical therapy trials that can be applied by trial reviewers.

## Materials and methods

Search for appropriate trials to be tested and data extraction

We searched for RCTs that reported a negative Berger-Exner test result about their trial data. Since the Berger-Exner test has not yet been widely adopted, a search in PubMed using the search term “berger-exner test” did not yield any relevant trial citations. Instead, we searched Google Scholar until November 24, 2023, with the same search term, which yielded 108 citations. Of these, a total of eight suitable RCTs [9-16] were identified.

We matched these eight RCTs with eight prospective controlled COHORT studies [17-24] as positive controls. The studies were selected by searching PubMed until November 26, 2023, using the search term: “prospective controlled COHORT study”, sorted by: Publication Date. A total of 150 citations were identified. From these, the first eight listed studies were selected that fulfilled all of the following selection criteria: The term “prospective controlled COHORT study” was included in the article title and the baseline variable “age” was reported for two study groups, including mean value, standard deviation (SD) and number of patients.

We extracted the mean value (SD) for the baseline variable “age” and patient number for test and control groups from all 16 articles. Where more than one test group was reported, we selected the first group listed for data extraction. The total age range for patients of the two groups combined was also extracted when reported. If the range was not reported, a reasonable estimate of the range was made.

Generation of simulated comparator trials (SCTs)

For each of the 16 studies, two SCTs were generated. Each SCT consisted of three parallel data columns entered into an MS Excel sheet (Microsoft® Corp., Redmond, WA, USA):

· Column 1: Ascending list of integers (1,2,3, … ), serving as patient ID;

· Column 2: Random allocation sequence for two groups, A and B;

· Column 3: List of randomly selected values within the trial-specific age range, sorted in ascending order.

The number of patients combined for the test and control group that were extracted from the test trial defined the length of all three columns. The random allocation sequence in column 2 was generated by block randomisation with block size 4 using the “Sealed Envelope” online tool [25]. The ascending list of randomly selected values in column 3 was generated using an online random number generator [26]. The comprehensive version of the online generator was used for randomly selecting the values of the baseline variable for each subject with the following settings: Lower/Upper limit as per age range; Number to be generated = Total number of patients, combined for the trial test and control group; Allow duplication of results? = Yes; Sort the results? = Ascend; Type of result to generate = Integer.

Trial testing for selection bias risk

**Figure 1:**
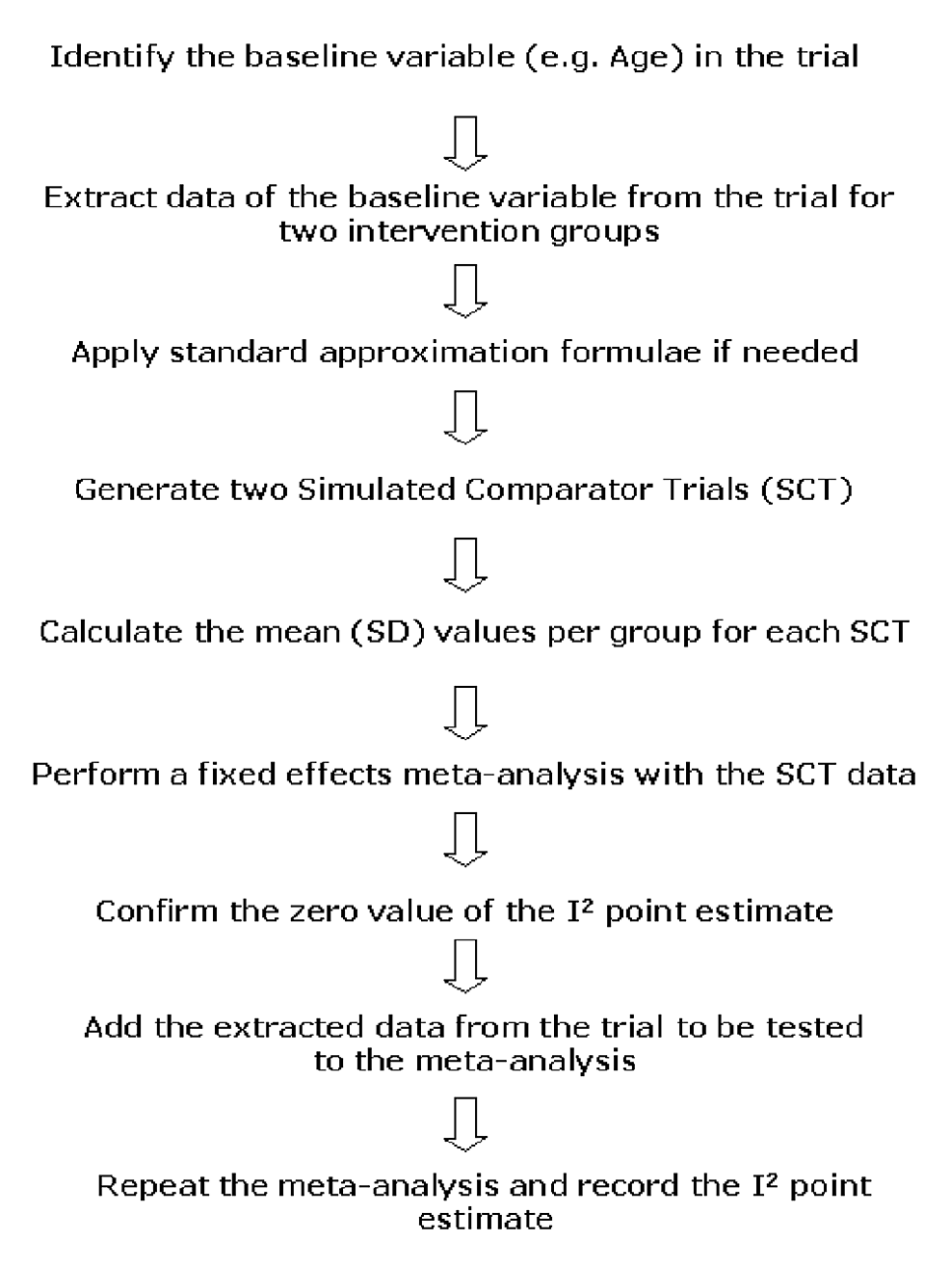
Summary of the applied test method

The three generated data columns were sorted according to allocation to groups A and B in column 2 using the sorting function in MS Excel. After sorting, the mean (SD) together with the sample size per groups A and B for both SCTs was calculated and entered into a fixed effect meta-analysis (Review Manager - RevMan 5.0.24 software; The Cochrane Collaboration, Oxford, UK). The two SCTs were pooled using the inverse variance method and the resulting zero I2 point estimate was confirmed. As the next step, the mean age (SD) together with the sample size per group that was extracted from the study to be tested was also entered and the meta-analysis was repeated. The resulting new I2 point estimate was recorded. All steps of the applied test method are summarised in Figure 1 and were conducted separately for each of the eight RCTs and eight cohort studies (see Appendices).

If the I^2^ point estimate of the repeated meta-analysis was also zero, the test result was considered negative and no selection bias risk for the tested study was assumed. If the point estimate showed an I^2^ > 0% value, the test result was considered positive and the tested study was assumed to be at risk of selection bias.

## Results

SCT generation assured perfect, albeit artificially ideal, random allocation of the simulated baseline values to groups A and B. Therefore, the pooling of the two SCTs in a baseline data meta-analysis yielded zero heterogeneity that was reflected as I^2^ = 0. The zero heterogeneity between the two SCTs thus served as an ideal comparator, against which the group distribution of the baseline value, age, from the trial to be tested (RCT or cohort study) was compared.

The test yielded negative results for all RCTs (Table 1) and positive results for six [17-21,23] out of the eight cohort studies (Table 2). All generated meta-analyses for both cohort studies and RCTs are presented in the Appendices section.

**Table 1:** Tested RCTs with reported negative Berger-Exner test results *Higher range limit estimate; **Age in months; NT: combined number of patients; SD: standard deviation; N: patient number per group; SCT: simulated comparator trial; Test result 0/1: negative/positive; bias risk N/Y: No/Yes

Test trial ref.	Test trial/extracted baseline variable (age in years)								SCT 1 - Variable						SCT 2 - Variable						I^2 ^(%)		Test result (0/1)	Bias risk (N/Y)
	Range	N_T_	Test group			Control group			Group A			Group B			Group A			Group B			SCT 1,2	SCT 1,2 and Test trial		
			Mean	SD	N	Mean	SD	N	Mean	SD	N	Mean	SD	N	Mean	SD	N	Mean	SD	N				
[9]	75-95*	82	81.30	4.80	41	81.30	4.80	41	84.58	6.20	41	84.68	6.20	41	84.46	5.82	41	84.44	5.93	41	0	0	0	N
[10]	13-21	418	17.30	2.10	211	17.10	2.10	207	17.02	2.42	209	17.01	2.42	209	17.04	2.61	209	17.03	2.60	209	0	0	0	N
[11]	21-50	71	36.00	8.00	37	35.00	8.00	34	34.44	8.16	36	34.20	7.52	35	35.42	8.84	36	35.17	8.40	35	0	0	0	N
[12]	4-6**	95	5.40	0.50	49	5.60	0.50	46	5.04	0.82	48	5.06	0.81	47	4.98	0.90	48	4.98	0.89	47	0	0	0	N
[13]	18-80*	683	29.90	7.00	344	30.20	7.00	339	49.36	18.07	341	49.44	18.12	342	47.90	18.23	341	47.98	18.30	342	0	0	0	N
[14]	18-80*	26	40.00	11.60	13	34.00	10.90	13	45.64	18.29	14	40.00	15.81	12	54.50	18.55	14	47.17	16.92	12	0	0	0	N
[15]	19-47	31	23.68	2.72	16	25.13	5.50	15	32.12	7.68	16	31.40	8.16	15	30.88	7.34	16	30.33	7.72	15	0	0	0	N
[16]	18-80*	167	57.94	15.83	83	59.48	15.53	84	47.94	18.78	83	48.43	19.00	84	48.39	18.75	83	48.86	18.94	84	0	0	0	N

**Table 2:** Tested cohort studies *Higher range limit estimate; NT: combined number of patients; SD: standard deviation; N: patient number per group; SCT: simulated comparator trial; Test result 0/1: negative/positive; bias risk N/Y: No/Yes

Test trial Ref.	Test trial/extracted baseline variable (age in years)								SCT 1 - Variable						SCT 2 - Variable						I^2 ^(%)		Test result (0/1)	Bias risk (N/Y)
	Range	N_T_	Test group			Control group			Group A			Group B			Group A			Group B			SCT 1, 2	SCT 1, 2 and test trial		
			Mean	SD	N	Mean	SD	N	Mean	SD	N	Mean	SD	N	Mean	SD	N	Mean	SD	N				
[17]	18-80*	61	64.22	12.00	31	50.73	14.00	30	49.39	18.64	31	48.03	17.93	30	51.10	17.94	31	50.03	17.74	30	0	71	1	Y
[18]	18-80*	282	40.80	12.50	142	44.80	12.30	140	48.49	18.11	141	48.51	18.11	141	49.42	17.41	141	49.45	17.52	141	0	44	1	Y
[19]	18-80*	81	45.17	12.96	30	50.75	10.27	51	46.15	16.88	41	45.60	16.75	40	49.54	19.66	41	48.98	19.34	40	0	17	1	Y
[20]	25-80*	98	36.80	8.80	49	41.90	9.20	49	51.49	15.98	49	51.63	16.27	49	55.33	16.14	49	55.43	16.36	49	0	30	1	Y
[21]	18-80*	53	49.00	17.00	12	59.00	14.00	41	50.44	17.21	27	48.85	16.94	26	50.89	19.12	27	49.65	18.33	26	0	37	1	Y
[22]	8-18	47	14.10	2.30	30	14.30	3.10	17	12.78	2.62	23	13.04	2.94	24	12.56	3.27	23	12.75	3.46	24	0	0	0	N
[23]	19-88	307	45.30	10.60	164	53.40	12.4	143	53.09	21.34	153	53.21	21.49	154	52.72	19.28	153	53.00	19.53	154	0	86	1	Y
[24]	8-18	52	13.84	2.50	32	14.36	3.20	20	13.00	2.95	26	12.88	2.89	26	12.58	2.83	26	12.46	2.72	26	0	0	0	N

## Discussion

In this article, a quantitative method is proposed for identifying potential selection bias in prospective controlled clinical therapy trials that can be applied by trial reviewers not involved in the trial conduct. Like the method proposed by Hicks et al. for identifying selection bias in meta-analyses [6], our method also relies on using the I^2^ point estimate as an indicator for heterogeneity and subsequent imbalances of patient baseline characteristics between trial intervention groups.

The use of the I^2^ point estimate for correctly reflecting heterogeneity has been criticized as being susceptible to confounding by trial number and the sample size of the trials included in a meta-analysis [8,27]. However, these concerns may not be relevant to meta-analyses of baseline data and Mickenautsch and Yengopal found no effect of both, trial number and trial sample size, on the accuracy of the test proposed by Hicks et al. [6,28].

Unlike the method by Hicks et al., the data of only one actual trial are entered into a fixed effect meta-analysis, while the data of the two SCTs are ideal simulation constructs with the aim to represent a zero I^2^ point estimate against which the data of the actual trial are tested. Therefore, the need for calculating the t-statistics per trial, as proposed by Hicks, is not needed, because any changes in the I^2^ value from zero can be solely ascribed to the tested trial.

For our test, we used only “age” as the baseline variable. Clarke et al. found “age” to be a good predictor for outcome, an easy variable to reflect patient misallocation, and also observed that most trials report the mean (SD) of patients’ age per group and thus appears to be the most available baseline variable for testing [7]. However, Hicks et al. recommended the use of more than one baseline variable for bias testing in order to increase test precision. Hence, in praxis, our test method may benefit from using other reported variables as well.

To demonstrate the accuracy of our test method, we exclusively selected RCTs with negative Berger-Exner tests as negative controls. Our test results fully mirrored the reported negative results of the highly accurate Berger-Exner test in all available eight trials (Table 1). We further chose to test the same number of prospective controlled cohort studies as positive controls. Cohort studies are observational studies, which do not use random allocation of patients into study groups. Due to the lack of randomisation, a high chance of an uneven distribution of the baseline variable “age” and thus high baseline heterogeneity in cohort studies was expected. According to expectation, our test yielded positive results in six out of the eight studies (Table 2). However, an even distribution of “age” between groups in cohort studies may always be possible by chance. Accordingly, our test yielded negative results in two studies [22,24]. A detailed reading of the study report by King et al. [24] established the possibility that groups were matched by age, which would have contributed to a zero-baseline heterogeneity and thus a negative test result.

Notwithstanding, our test method yielded mostly expected results with both types of study and therefore a reasonably high accuracy of our method for correctly identifying selection bias risk is suggested.

Limitations and recommendations for authors

The main limitation of our investigation is due to the small number of RCTs with negative Berger-Exner tests that could be found. Unfortunately, the test is not yet widely adopted. However, because of its high accuracy, the inclusion of the test into an RCT provided us with a comparator of almost absolute certainty. Further information about the accuracy of our test method may be achieved in the form of a simulation study where biased together with non-biased trials are simulated and then tested in a sufficiently large number, determined by sample size calculation according to the method by Buderer et al. [29]. Such investigation might be able to establish the sensitivity and specificity of our test method with higher precision.

To avoid any uncertainties during the investigation of our novel method, we included only studies that reported mean baseline values with SD. However, in practice, reviewers may use an approximation formula when only the median and range are reported instead [30] and, if needed, convert the reported standard error into SD [31].

Unlike the use of the I^2^ point estimate for testing bias risk in meta-analyses [6], our method can establish only whether high selection bias risk is likely or not. It does not provide the possibility to establish whether such bias risk has affected the trial outcome in terms of effect magnitude and effect direction. Instead, a positive test result may provide an empirical basis for rating a trial as of high risk of bias in the bias domain “bias arising from the randomisation process” when using the RoB 2 tool [2] or as “falsified” at corroboration level 2 when using the CQS-2B [3].

## Conclusions

All test results remained within the expected limits for both study types, suggesting a reasonably high accuracy for correctly identifying selection bias risk. However, the method does not provide the possibility to establish whether such bias risk has actually altered trial outcomes. Instead, a positive test result may provide an empirical basis for rating a trial as of high selection bias risk during trial appraisal.

Preprint option

This manuscript has been made available online as preprint in Research Square (www.researchsquare.com/): Mickenautsch S, Yengopal Y. A test method for identifying selection bias risk in prospective controlled clinical therapy trials using the I2 point estimate (Preprint), 13 December 2023, PREPRINT (Version 1) available at Research Square (https://doi.org/10.21203/rs.3.rs-3748643/v1).

## Appendices

All data are fully available without restriction via: https://data.mendeley.com/datasets/px8wv4bw95/
